# Conductance Changes of Na^+^ Channels during the Late Na^+^ Current Flowing under Action Potential Voltage Clamp Conditions in Canine, Rabbit, and Guinea Pig Ventricular Myocytes

**DOI:** 10.3390/ph16040560

**Published:** 2023-04-07

**Authors:** Balázs Horváth, Zsigmond M. Kovács, Csaba Dienes, József Óvári, Norbert Szentandrássy, János Magyar, Tamás Bányász, András Varró, Péter P. Nánási

**Affiliations:** 1 Department of Physiology, Faculty of Medicine, University of Debrecen, 4032 Debrecen, Hungary; 2 Department of Basic Medical Sciences, Faculty of Dentistry, University of Debrecen, 4032 Debrecen, Hungary; 3 Division of Sport Physiology, Department of Physiology, Faculty of Medicine, University of Debrecen, 4032 Debrecen, Hungary; 4 Department of Pharmacology and Pharmacotherapy, Faculty of Medicine, University of Szeged, 6720 Szeged, Hungary; 5 Department of Dental Physiology and Pharmacology, Faculty of Dentistry, University of Debrecen, 4032 Debrecen, Hungary

**Keywords:** late Na^+^ current, ventricular repolarization, action potential voltage clamp, ventricular myocytes, mammalian heart

## Abstract

Late sodium current (I_Na,late_) is an important inward current contributing to the plateau phase of the action potential (AP) in the mammalian heart. Although I_Na,late_ is considered as a possible target for antiarrhythmic agents, several aspects of this current remained hidden. In this work, the profile of I_Na,late_, together with the respective conductance changes (G_Na,late_), were studied and compared in rabbit, canine, and guinea pig ventricular myocytes using the action potential voltage clamp (APVC) technique. In canine and rabbit myocytes, the density of I_Na,late_ was relatively stable during the plateau and decreased only along terminal repolarization of the AP, while G_Na,late_ decreased monotonically. In contrast, I_Na,late_ increased monotonically, while G_Na,late_ remained largely unchanged during the AP in guinea pig. The estimated slow inactivation of Na^+^ channels was much slower in guinea pig than in canine or rabbit myocytes. The characteristics of canine I_Na,late_ and G_Na,late_ were not altered by using command APs recorded from rabbit or guinea pig myocytes, indicating that the different shapes of the current profiles are related to genuine interspecies differences in the gating of I_Na,late_. Both I_Na,late_ and G_Na,late_ decreased in canine myocytes when the intracellular Ca^2+^ concentration was reduced either by the extracellular application of 1 µM nisoldipine or by the intracellular application of BAPTA. Finally, a comparison of the I_Na,late_ and G_Na,late_ profiles induced by the toxin of *Anemonia sulcata* (ATX-II) in canine and guinea pig myocytes revealed profound differences between the two species: in dog, the ATX-II induced I_Na,late_ and G_Na,late_ showed kinetics similar to those observed with the native current, while in guinea pig, the ATX-II induced G_Na,late_ increased during the AP. Our results show that there are notable interspecies differences in the gating kinetics of I_Na,late_ that cannot be explained by differences in AP morphology. These differences must be considered when interpreting the I_Na,late_ results obtained in guinea pig.

## 1. Introduction

The late Na^+^ current (I_Na,late_) is a depolarizing current of ventricular cardiomyocytes [1,2,3] with important pathophysiological roles [4,5,6,7,8]. I_Na,late_ contributes to the plateau phase of the ventricular action potential (AP) and is responsible for substantial Na^+^ entry through sodium channels [9,10,11]. Augmentation of I_Na,late_ leads to increased arrhythmia propensity associated with a longer action potential, increased heterogeneity of repolarization, and the development of afterdepolarizations [5,12,13,14,15]. Consequently, selective inhibition of I_Na,late_ has become a realistic therapeutical strategy [9,16,17,18,19,20,21].

Many biophysical mechanisms contribute to I_Na,late_. At first, it was described by the overlap of the steady-state activation and inactivation of the Na^+^ current (the so-called “window Na^+^ current”) [22,23]. Later, the gating kinetics of cardiac Na^+^ channels (burst mode and late scattered openings) were added to the picture [4,6]. Despite its pathophysiological importance, some characteristics of I_Na,late_ are still quite obscure.

I_Na,late_ has been studied in a variety of mammalian cardiac tissues including rabbit [24], guinea pig [25,26], porcine [27], canine [7,26,28,29,30], and human [8,14,26,31,32] cardiomyocytes. Most of the available experimental data on I_Na,late_ originates from conventional voltage clamp, which is a method that is not able to visualize the actual I_Na,late_ flowing under a real AP. One possibility of resolving this problem is the action potential voltage clamp technique (APVC), which delivers the cell’s own AP, or a “canonical” AP as a command signal. Using this method, it is possible to display the actual current flowing under the command AP voltage pulse. However, only a limited number of such studies are available [25,26,29,30,33]. In addition, most of these data are restricted to recordings of I_Na,late,_ without giving insights into the actual conductance changes (G_Na,late_) during the AP.

In a previous study, we studied and compared the profile of I_Na,late_ in canine, human, and guinea pig myocytes [26]. In that study, no conductance analysis was performed, therefore, it was not possible to separate the contribution of interspecies differences in the Na^+^ channel gating kinetics and the consequences of changes in the driving force during the differing AP contours. Therefore, in this study, the current profiles (I_Na,late_), together with the underlying conductance changes (G_Na,late_), were monitored throughout the action potential under self APVC conditions and compared in ventricular myocytes obtained from three frequently used mammalian species: dog, rabbit, and guinea pig. Application of the APVC technique offers the possibility of studying the currents under physiological conditions, while the inclusion of G_Na,late_ in the analysis offsets the driving force related changes in the current during the AP, allowing for some insights into channel gating. The results indicate profound differences in both the I_Na,late_ and G_Na,late_ between guinea pig and canine or rabbit currents, which may reflect genuine interspecies differences in Na^+^ channel gating rather than differences in the action potential morphologies. These differences must be considered when interpreting I_Na,late_ results obtained in various species.

## 2. Results

**Figure 1:**
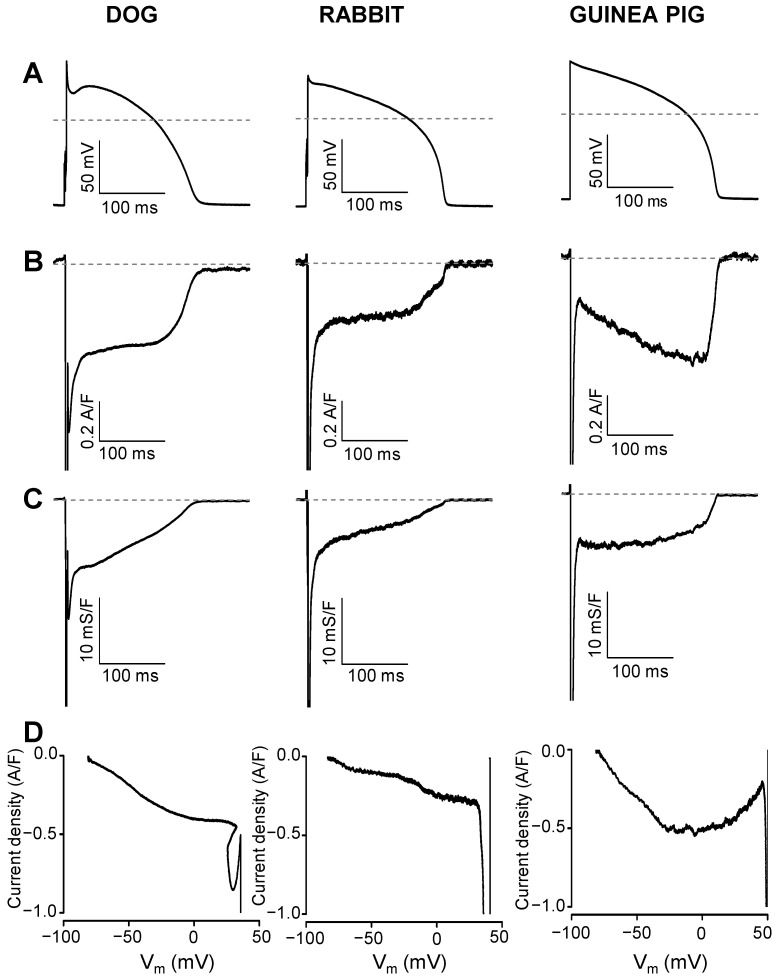
Representative I_Na,late_ (**B**) and G_Na,late_ (**C**) profiles recorded under self APVC conditions from the canine, rabbit, and guinea pig ventricular myocytes. I_Na,late_ was dissected as a 10 µM TTX-sensitive current using the own AP of the cell as a command signal (**A**). The current–voltage relationships (phase–plane trajectories) are displayed in panel (**D**).

**Figure 2:**
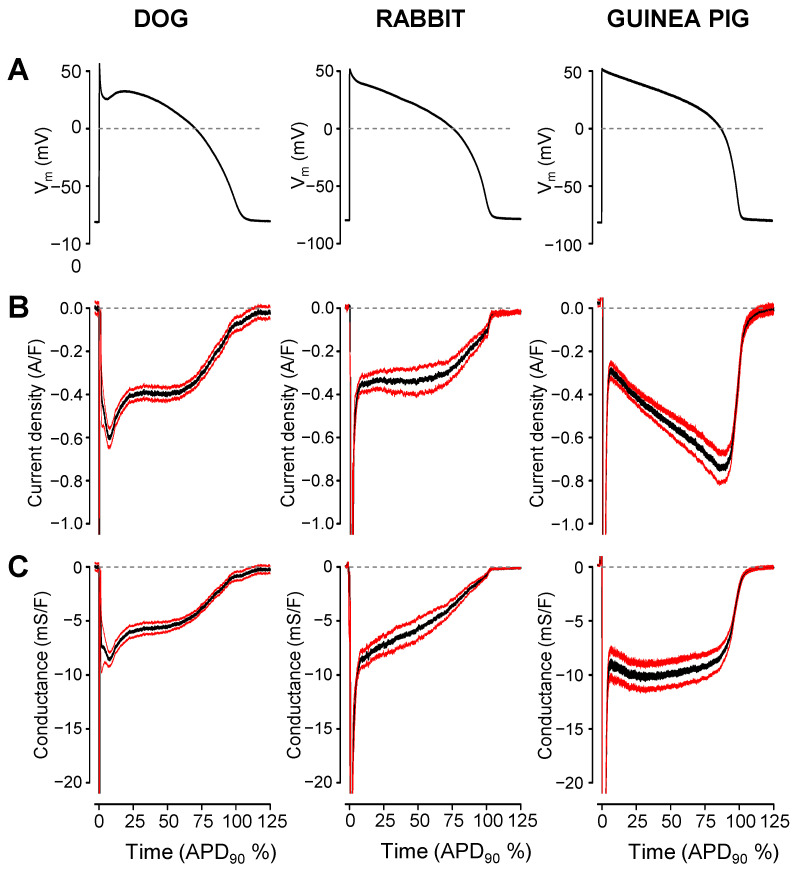
Average I_Na,late_ (**B**) and G_Na,late_ (**C**) profiles recorded under self APVC conditions from 15 canine, six rabbit, and 18 guinea pig ventricular myocytes. Black lines are the mean data, while red ones represent the SEM values. For the sake of comparability, the I_Na,late_ and G_Na,late_ values were averaged according to their positions on the time axis, where the action potential duration measured at 90% repolarization (APD_90_) was considered as 100%. Representative command APs are shown in the top row (**A**).

**Figure 3:**
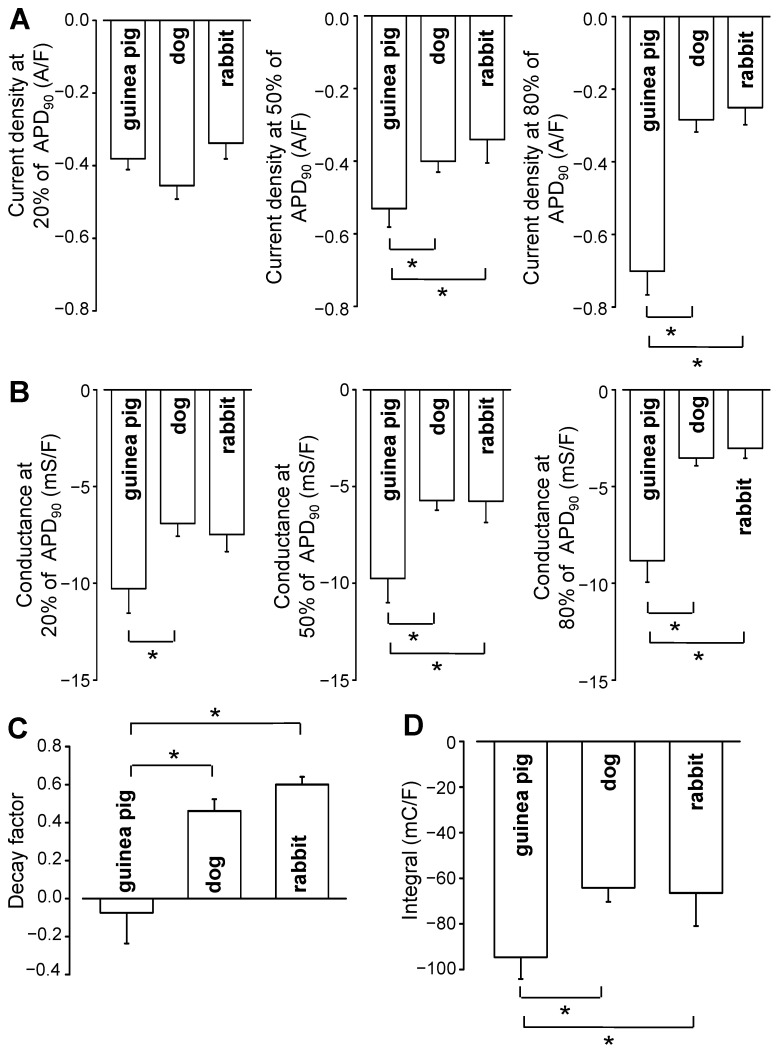
Average I_Na,late_ (**A**) and G_Na,late_ (**B**) values measured at 20%, 50%, and 80% of APD_90_ in the canine, rabbit, and guinea pig myocytes, respectively. Decay factors (defined as G_20%_ − G_80%_)/G_20%_) (**C**) and current integrals (**D**) obtained for 15 canine, six rabbit, and 18 guinea pig myocytes. Asterisks (*) denote significant differences (*p* < 0.05) determined using the Student’s *t*-test for unpaired data.

Representative I_Na,late_ and G_Na,late_ profiles obtained in canine, rabbit, and guinea pig ventricular myocytes are displayed in Figure 1. Currents were recorded under self APVC conditions (i.e., the cell’s own AP was used as a command pulse in all cases) (Figure 1A). As shown in Figure 1B,C, the density of I_Na,late_ was relatively constant during the AP plateau phase and decreased only along the terminal repolarization of the AP in canine and rabbit myocytes, while G_Na,late_ decreased monotonically in these cells during the AP. In contrast, in guinea pig myocytes, I_Na,late_ increased monotonically, while G_Na,late_ remained largely unchanged during the AP plateau. The current–voltage relationships were also different in shape in the guinea pig comparing to dog or rabbit (see phase–plane trajectories in Figure 1D). These characteristic interspecies differences in the I_Na,late_ and G_Na,late_ profiles are demonstrated statistically in the graphs of Figure 2, where the black curves represent the average, while the red ones indicate the SEM values. To make the individual results comparable, the I_Na,late_ and G_Na,late_ values were averaged according to their positions on the time axis, where the action potential duration measured at 90% repolarization (APD_90_) was considered as 100%. Accordingly, as seen previously, canine and rabbit I_Na,late_—and more prominently G_Na,late_—showed a “decrescendo” profile (i.e., their amplitudes decreased from the upstroke to phase 3 repolarization), contrary to guinea pig cells, where the amplitude of I_Na,late_ monotonously increased along the AP plateau (“crescendo”) and declined only in the terminal repolarization phase (Figure 2B,C). G_Na,late_, however, was quite stable during the entire plateau in guinea pig. The average I_Na,late_ and G_Na,late_ values measured at 20%, 50%, and 80% of APD_90_ in the canine, rabbit, and guinea pig myocytes are presented in Figure 3A,B, respectively. The conductance changes demonstrated in Figure 1 and Figure 2 suggest that the rate of decay of G_Na,late_ during the AP may be faster in canine and rabbit myocytes than in the guinea pig cells. The rate of decay of G_Na,late_ was estimated as the reduction in G_Na,late_ between the time spent between 20% and 80% of APD_90_, normalized to G_Na,late_, which was measured at the time of 20% of APD_90_ (“decay factor”: defined as (G_20%_ − G_80%_)/G_20%_). Indeed, this decay factor was significantly less in the guinea pig (−0.07 ± 0.16, n = 18) than in canine (0.46 ± 0.06, n = 15) or rabbit (0.60 ± 0.04, n = 6) myocytes (Figure 3C). Although the decay of G_Na,late_ was seemingly faster in rabbits than in dogs, this difference was not significant statistically. The charge carried by I_Na,late_ (i.e., the integrals) was similar in dog (−64.2 ± 6 mC/F) and rabbit (−66.5 ± 14.6 mC/F), but both of them were significantly less than the integral obtained in guinea pig cells (−94.6 ± 9.6 mC/F), as displayed in Figure 3D.

**Figure 4:**
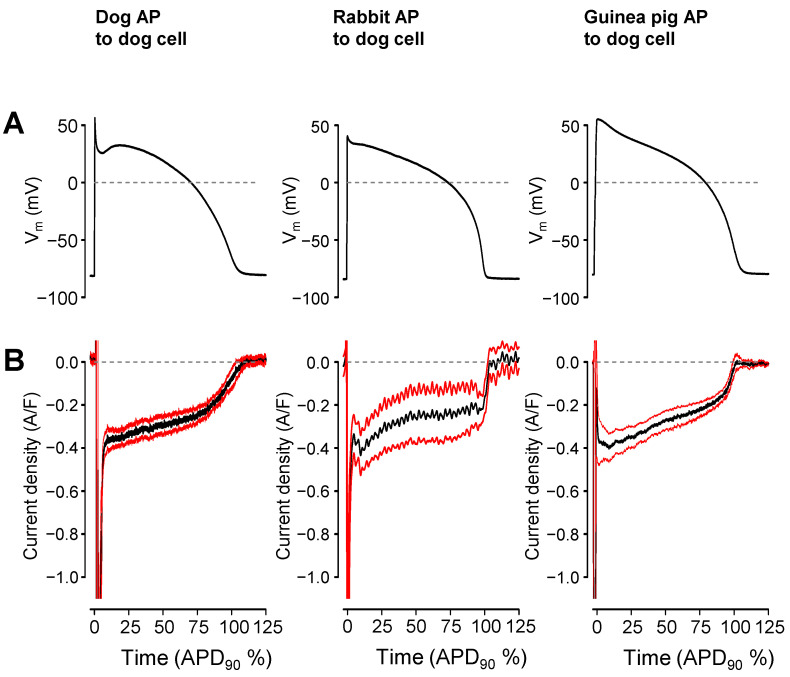
Effect of the shape of the command AP on the profile of I_Na,late_ in the canine myocytes. Black lines are the mean data, while red ones represent the SEM values. (**A**) The canonic command APs are shown in the top row. (**B**) I_Na,late_ profiles, averaged according to their positions on the time axis, where the action potential duration measured at 90% repolarization (APD_90_) was considered as 100%.

Current profiles under the APVC conditions are affected by the shape of the AP voltage pulse [34,35]. Therefore, time-matched canonic rabbit and guinea pig APs were delivered to canine myocytes to answer the question of whether the marked differences seen in the G_Na,late_ profiles in guinea pig versus dog and rabbit are related to differences in AP configuration or are genuine interspecies differences in Na^+^ channel gating [36]. During these experiments, 1 µM nisoldipine was present in the cell bath in order to diminish possible inhomogeneity originating from differences in the [Ca^2+^]_i_ levels of the individual cells. Despite applying rabbit or guinea pig command APs on canine cells, the shape of I_Na,late_ remained monotonically decreasing —the characteristic of canine I_Na,late_ (Figure 4). Importantly, the current integrals were similar in the canine cells independent of whether the canine, rabbit or guinea pig command APs were applied (canine AP: −48.5 ± 5 mC/F, n = 19; guinea pig AP: −49.9 ± 8.4 mC/F, n = 8; rabbit AP: −57.0 ± 22.2 mC/F, n = 4; N.S.).

**Figure 5:**
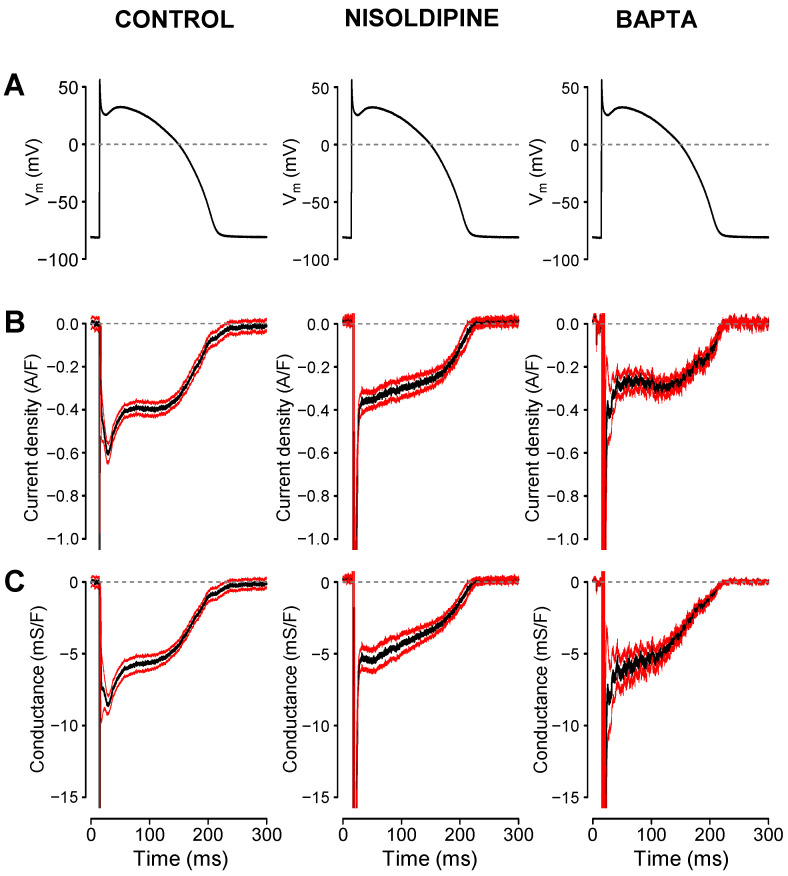
Effects of 1 µM extracellular nisoldipine (n = 19) and 10 mM intracellular BAPTA (n = 10) on the I_Na,late_ (**B**) and G_Na,late_ (**C**) profiles of the canine myocytes. The average control record was obtained from 11 cells. Black lines are the mean data, the red ones represent the SEM values. The I_Na,late_ and G_Na,late_ values were averaged according to their positions on the time axis, where the action potential duration measured at 90% repolarization (APD_90_) was considered as 100%. Command APs are shown in the top row (**A**).

To study the role of [Ca^2+^]_i_ in the regulation of I_Na,late_, the cytosolic Ca^2+^ was reduced by applying either 1 µM nisoldipine in the bathing medium or 10 mM BAPTA in the pipette solution. In the latter case, measurements started 10 min after rupturing the seal to let the Ca^2+^ chelator BAPTA equilibrate between the pipette solution and the intracellular space. The I_Na,late_ and G_Na,late_ profiles obtained with and without nisoldipine or BAPTA in the canine myocytes are presented in Figure 5. The amplitude of I_Na,late_ was reduced when the intracellular Ca^2+^ concentration was decreased by nisoldipine (−330 ± 30 mA/F vs. −457 ± 38 mA/F at 20% APD_90_, and −282 ± 38 vs. −412 ± 37 mA/F at 50% APD_90_, *p* < 0.05, n = 19 vs. n = 15), although at 80% APD_90_, the difference was not significant. The effect of BAPTA was significant at each segment of APD (−277 ± 44 vs. −457 ± 38 mA/F at 20%, −306 ± 36 vs. −412 ± 37 at 50%, and −223 ± 32 vs. −284 ± 34 mA/F at 80% APD_90_, *p* < 0.05, n = 11 vs. n = 15). When calculating the conductances, G_Na,late_ was reduced by nisoldipine, similarly to the reduction in I_Na,late_ (5.0 ± 0.5 mS/F vs. 6.9 ± 0.7 mS/F at 20% APD_90_, and 4.0 ± 0.5 vs. 5.9 ± 0.6 mS/F at 50% APD_90_), but the reduction in G_Na,late_ was significant only at 80% APD_90_ (2.0 ± 0.3 mS/F vs. 3.5 ± 0.4 mS/F). In other words, nisoldipine decreased G_Na,late_ more prominently at the initial, while BAPTA at the later segment of the AP. Current integrals, however, were significantly reduced by both nisoldipine and BAPTA (−48.5 ± 5 mC/F vs. −63.9 ± 6 mC/F and −46.7 ± 5 vs. −63.9 ± 6 mC/F, respectively).

**Figure 6:**
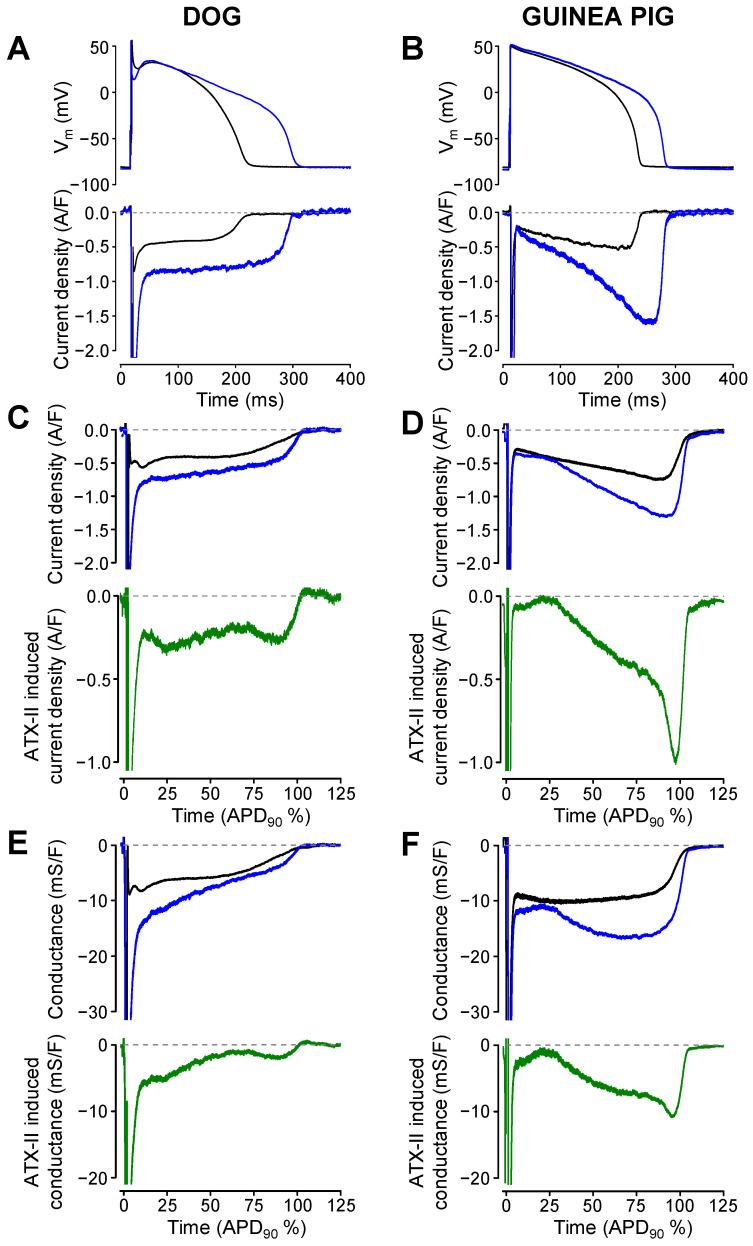
The effect of ATX-II on the TTX-sensitive current and conductance profiles in canine (**left**) and guinea pig (**right**) myocytes. (**A**,**B**) panels show representative APs and TTX-sensitive current records obtained before (black) and after (blue) the application of ATX-II (1 nM in dog and 10 nM in guinea pig). Average currents and conductances are shown using the same color code in panels (**C**–**F**). Average ATX-II induced currents (**C**,**D**) and conductances (**E**,**F**), obtained in six canine and four guinea pig myocytes, are displayed in a green color.

The toxin of *Anemonia sulcata* (Anemone toxin II, ATX-II) induces a current in cardiac tissues closely resembling I_Na,late_ by inhibiting the fast inactivation mechanism of Na^+^ channels [37,38]. Therefore, the effect of 10 nM ATX-II in guinea pig and 1 nM ATX-II in canine myocytes were studied (Figure 6). When 10 nM ATX-II was applied for 3 min, canine cells usually produced early afterdepolarizations (data not shown). To prevent these, canine myocytes were treated with only 1 nM ATX-II. This demonstrates that the sodium channels in canine myocytes are more sensitive to ATX-II exposure than the channels in guinea pig cells. ATX-II lengthened the AP and increased the amplitude of I_Na,late_ in both species (Figure 6A,B). Since I_Na,late_ was recorded in the absence and in the presence of ATX-II in different sets of experiments, only the average I_Na,late_ profiles (Figure 6C,D) and G_Na,late_ profiles (Figure 6E,F) could be compared (without SEM values). Although the ATX-II induced currents were not identical to native I_Na,late_, their profiles were similar in shape to the profiles of the native I_Na,late_ in both species. The ATX-II induced current displayed a “decrescendo” profile in the canine and “crescendo” profile in the guinea pig myocytes—just like their respective native I_Na,late_ profiles. However, in contrast to the native G_Na,late_ profile, which was largely constant during the AP plateau in guinea pig, the ATX-II induced conductance displayed a marked *increasing* tendency during the guinea pig AP (compare these changes with those demonstrated in Figure 1 or Figure 2 for G_Na,late_). Accordingly, the decay factor estimated for G_Na,late_ in the presence and absence of ATX-II was similar (0.54 ± 0.06, n = 6 and 0.46 ± 0.06, n = 15, respectively, N.S.) in the canine myocytes. In guinea pigs, the decay factor became significantly more negative in the presence of ATX-II than that measured under control conditions (−0.95 ± 0.81, n = 4 vs. −0.07 ± 0.16, n = 18, *p* < 0.05). These results demonstrate that the behavior of the ATX-II-induced conductance is markedly different from the native G_Na,late_ in guinea pig myocytes.

## 3. Discussion

Our study was the first to visualize the G_Na,late_ profiles in canine, rabbit, and guinea pig ventricular cells under self-APVC conditions. In the canine and rabbit myocytes, G_Na,late_ decreased monotonically during the AP plateau phase, while in guinea pig, the conductance was largely unchanged during the AP plateau and decreased abruptly at terminal repolarization only. This may explain why I_Na,late_ increased in guinea pig, while decreased in dog and rabbit during the plateau phase of the AP, as demonstrated in Figure 1 and Figure 2. The decay factor, used as an indicator of Na^+^ channel inactivation, was significantly greater for the rabbit or canine than for guinea pig cells, indicating that Na^+^ channels are likely largely inactivated by the time of terminal repolarization in rabbit and dog, in contrast to guinea pig. Consequently, the increased driving force arising during terminal repolarization can hardly increase the amplitude of I_Na,late_ in dog or rabbit, but can act in guinea pig to increase I_Na,late_. Indeed, the time constant of inactivation obtained for I_Na,late_ at −20 mV under conventional voltage clamp conditions was 2.5 times longer in the guinea pig than in canine myocytes [26].

For calculating G_Na,late_, the Na^+^ concentration of the pipette solution (6 mM) was used as the Na^+^ concentration on the intracellular side of the cell membrane. However, this is only an approximation, mainly because the Na^+^ concentration in the vicinity of the Na^+^ channels (subsarcolemmal space; [Na^+^]_subs_) can be quite different than the Na^+^ concentration of the bulk cytosol, caused by the dynamic transmembrane sodium influx and efflux mechanisms that are taking place here [39,40,41]. Additionally, even the Na^+^ concentration of the bulk cytosol undergoes substantial changes when the cells are paced [42,43]. Considering these factors, subsarcolemmal Na^+^ concentration can increase to around 8–9 mM in our paced cells. As [Na^+^]_subs_ increases, the equilibrium potential for Na^+^ decreases. A [Na^+^]_subs_ value of 8 mM would account for a reversal potential of +77.6 mV, instead of the +85.3 mV used in our calculations. A smaller reversal potential, by generating a smaller driving force for Na^+^ movement, would result in larger calculated Na^+^ conductances. As our [Na^+^]_subs_ approximation was the same for all three species, and we have no data showing marked differences in the [Na^+^]_subs_ dynamics in these three species, we likely systematically underestimated G_Na,late_ in all three species to a similar extent.

The monotonically increasing I_Na,late_ profile under the AP plateau in guinea pig myocytes could be explained by the non-equilibrium gating of the sodium channels as published by Clancy et al. [44]. According to this model, I_Na,late_ accumulates during the guinea pig AP plateau as a consequence of the slow, ramp-like repolarization in this phase. However, when guinea pig or rabbit APs were applied as command pulses onto canine myocytes, the I_Na,late_ profile remained similar to the native canine I_Na,late_. It must be also kept in mind that the morphology of rabbit and guinea pig APs are quite similar—in contrast to the spike-and-dome configuration of the canine AP—while their I_Na,late_ and G_Na,late_ profiles were drastically different. Therefore, we concluded that the slower inactivation kinetics of Na^+^ channels, combined with the effect of the increasing driving force during repolarization, may create an I_Na,late_ profile that monotonically increased under the AP plateau phase in guinea pig. As I_Na,late_ is partly generated by Na^+^ channels other than Nav1.5 [5,45,46], the exact distribution of these Na^+^ channel isoforms or their respective regulatory subunits may contribute to the differences observed in various species (dog and rabbit vs. guinea pig). Further studies are necessary to clarify the electrophysiological details underlying the different inactivation kinetics of I_Na,late_ detected in different species.

The dissimilar I_Na,late_ profile in various species likely results in a different relative contribution of I_Na,late_ to the actual AP morphology as the APD changes. In guinea pigs, with a monotonically increasing I_Na,late_, the contribution of the current is likely to increase larger as the APD lengthens. Because the current has the biggest density around the final repolarization, any given APD lengthening effect, for example, the presence of a K^+^ channel inhibitor, will result in a larger extra inward current due to I_Na,late_. Consequently, the original APD prolonging effect may be stronger because of this mechanism. Furthermore, the larger Na^+^ and Ca^2+^ load due to the increasing I_Na,late_ results in an increased risk of arrhythmias.

In contrast, in dogs and rabbits, with a monotonically decreasing I_Na,late_, the contribution of I_Na,late_ becomes smaller at longer APDs. Since the current is very small at the terminal repolarization in rabbit and canine preparations, changes in APD will only have a very small effect on the Na^+^ efflux via I_Na,late_.

Similarly, the effects of I_Na,late_ blockers (e.g., APD shortening, cellular Na^+^ and Ca^2+^ load reduction) are likely to be smaller in rabbit or canine cardiac myocytes than in guinea pig cells. This must be considered when interpreting electrophysiological and pharmacological I_Na,late_ studies where a guinea pig model has been used. These problems are not anticipated when using canine or rabbit myocytes, since human I_Na,late_ also shows a “decrescendo” profile under APVC conditions, similar to canine and rabbit myocytes [26].

The present study also showed that the ATX-II-induced G_Na,late_ profile in guinea pig cells, under APVC conditions, had a substantially different shape than the native G_Na,late_, as the conductance associated with the ATX-II-induced current increased throughout the time course of the plateau phase of the AP—again, contrasting the respective conductance observed in canine myocytes (compare Figure 6E,F). This monotonic increase in the ATX-II-induced G_Na,late_ during the guinea pig AP was unexpected and may reflect the binding of ATX-II to the Na^+^ channels during the AP, however, in this case, it should have also been present in canine myocytes, which was clearly not observed. Or, alternatively, it may be explained by the proposed non-equilibrium gating of Na^+^ channels [44]. At present, we cannot distinguish between these possibilities. ATX-II is known to retard the inactivation of Na^+^ channels [37] and is utilized as an experimental tool to mimic augmented I_Na,late_ under pathological conditions [5]. This procedure might be misleading in guinea pig myocytes because of the pronounced differences between profiles of the native I_Na,late_ and the current induced by ATX-II. Because of this substantial difference in the current profile, the binding of ATX-II may also change the drug-sensitivity of the native guinea pig Na^+^ channels, causing difficulties in translating the experimental data collected in guinea pig preparations in the presence of ATX-II.

In summary, we can conclude that using guinea pig myocytes for drug studies related to the modification of I_Na,late_ may not be straightforward to translate to humans. Further detailed studies are required to describe the exact details of the gating kinetics of I_Na,late_ in various mammalian species.

## 4. Methods

### 4.1. Animals

All animal procedures conformed to the guidelines from Directive 2010/63/EU of the European Parliament on the protection of animals used for scientific purposes and to the protocol approved by the local Animal Care Committee (license No: 2/2020/DEMÁB). Adult mongrel dogs of either sex were anesthetized with intramuscular injections of 10 mg/kg ketamine hydrochloride (Calypsol, Richter Gedeon, Budapest, Hungary) + 1 mg/kg xylazine hydrochloride (Sedaxylan, Eurovet Animal Health BV, Bladel, The Netherlands). Single canine myocytes were obtained using the segment perfusion technique. Male guinea pigs and New Zealand white rabbits were heparinized and anesthetized with nembutal (100 mg/kg i.p.). After achieving deep anesthesia, the hearts were rapidly removed and mounted on a Langendorff apparatus allowing for retrograde perfusion of the aorta.

### 4.2. Isolation of Cardiomyocytes

Single canine myocytes were obtained by enzymatic dispersion using the segment perfusion technique, as previously described [47]. A wedge-shaped section of the ventricular wall supplied by the left anterior descending coronary artery was cannulated, dissected, and perfused with a Ca^2+^-free Joklik medium (M0518, Sigma-Aldrich, St. Louis, MO, USA) for 5 min. This was followed by a 30-min-long perfusion with Joklik medium supplemented with 1 mg/mL collagenase (Type II, Worthington Biochemical Co., Lakewood, NJ, USA; representing final activity of 224 U/mL) and 0.2% bovine serum albumin (Fraction V., Sigma-Aldrich) containing 50 µM Ca^2+^. After this, the normal external Ca^2+^ concentration was gradually restored and cells were stored in Eagle’s MEM (M0643, Sigma-Aldrich) until use.

Guinea pig and rabbit ventricular cells were obtained using a standard retrograde perfusion technique as previously described [25]. After mounting the aorta on a Langendorff device, the heart was washed with oxygenized Tyrode solution for 5 min and a further 3 min with Ca^2+^-free Tyrode solution to stop the heart. This perfusate was supplemented with 0.6 mg/mL collagenase (Type II, Worthington) and 0.05 mg/mL protease (Type XIV, Sigma-Aldrich). After this procedure, the left ventricle was minced into tissue chunks that were further incubated with enzyme solution for approximately 1 h. The normal external Ca^2+^ concentration was restored after harvesting the cells. The chemicals used in the experiments were obtained from Merck (Darmstadt, Germany; previously Sigma-Aldrich) unless otherwise stated.

### 4.3. Electrophysiology

Viable cells were placed in a 1 mL volume plexiglass chamber and continuously superfused with a modified Tyrode solution supplied by gravity flow at a rate of 1–2 mL/min. The modified Tyrode solution contained (in mM): NaCl 121, KCl 4, CaCl_2_ 1.3, MgCl_2_ 1, HEPES 10, NaHCO_3_ 25, glucose 10 at pH = 7.35. Osmolarity of the modified Tyrode solution was 300 ± 3 mOsm and the temperature was set to 37 °C. Experiments were performed under an inverted microscope placed on an anti-vibration table. Electrical signals were recorded with intracellular amplifiers (MultiClamp 700A or 700B, Molecular Devices, San Jose, CA, USA) and recorded with pClamp 10 software (Molecular Devices) after analogue–digital conversion (Digidata 1440A or 1332, Molecular Devices). Electrodes were fabricated from borosilicate glass with tip resistances of 2–3 MΩ after filling with pipette solution. The regular pipette solution contained (in mM): K-aspartate 120, KCl 30, MgATP 3, HEPES 10, Na_2_-phosphocreatine 3, EGTA 0.01, cAMP 0.002, KOH 10 at pH = 7.3. The osmolarity of the pipette solutions was 285 mOsm. Membrane currents were recorded using the whole-cell configuration of the patch-clamp technique. After establishing a high (1–10 GΩ) resistance seal by gentle suction, the cell membrane beneath the tip of the electrode was disrupted by further suction or by applying 1.5 V electrical pulses for 1 ms. The series resistance was typically 4–8 MΩ. Experiments were discarded when the series resistance changed substantially during the measurement.

APVC experiments were conducted according to the methods described previously [25,30,34,35]. In most experiments, the cell’s own AP was used as the command voltage (self APVC) at a pacing cycle length of 700 ms. In other experiments, a previously recorded “canonic” midmyocardial action potential (with average parameters and configuration) was applied to the voltage clamped canine cells as a command signal (canonic APVC). Current traces were recorded continuously under reference conditions and after 5 min superfusion with the specific Na^+^ channel inhibitor tetrodotoxin (TTX, 10 µM). I_Na,late_ was defined as a TTX-sensitive current, obtained by subtracting the post-TTX traces from the reference traces. During the analysis of I_Na,late_, the initial 15 ms after the AP upstroke was excluded from evaluation to omit the early Na^+^ current peak. To offset trace-to-trace fluctuations and to reduce noise, 20 consecutive I_Na,late_ traces were averaged, and the averaged curve was used for later analysis. I_Na,late_ was normalized to cell capacitance, determined in each cell by applying hyperpolarization from +10 to −10 mV for 15 ms. Conductance values associated with I_Na,late_ (G_Na,late_) were calculated by dividing the I_Na,late_ values by the driving force for Na^+^, defined as the difference in the actual transmembrane potential and Na^+^ equilibrium potential, estimated as +85.3 mV ([Na^+^]_o_ = 146 mM, [Na^+^]_i_ = 6 mM, T = 310 K).

### 4.4. Statistics

The results are expressed as mean ± SEM values, n denotes the number of myocytes studied. The statistical significance of differences was evaluated using one-way ANOVA followed by the Student’s *t*-test. Differences were considered significant when *p* was less than 0.05.

## Data Availability

Data is contained within the article.

## Abbreviations

I_Na,late_: late sodium current; AP: action potential, APVC: action potential voltage clamp, TTX: tetrodotoxin, ATX-II: *Anemonia sulcata* toxin II.
